# Supplementary figures and images for: mmu-miRNA-342-3p promotes hepatic stellate cell activation and hepatic fibrosis induced by Echinococcus multilocularis infection via targeting Zbtb7a
Source: PLoS Negl Trop Dis. 2023 Jul 25;17(7):e0011520. doi: 10.1371/journal.pntd.0011520 (PMC10403128; doi:10.1371/journal.pntd.0011520)

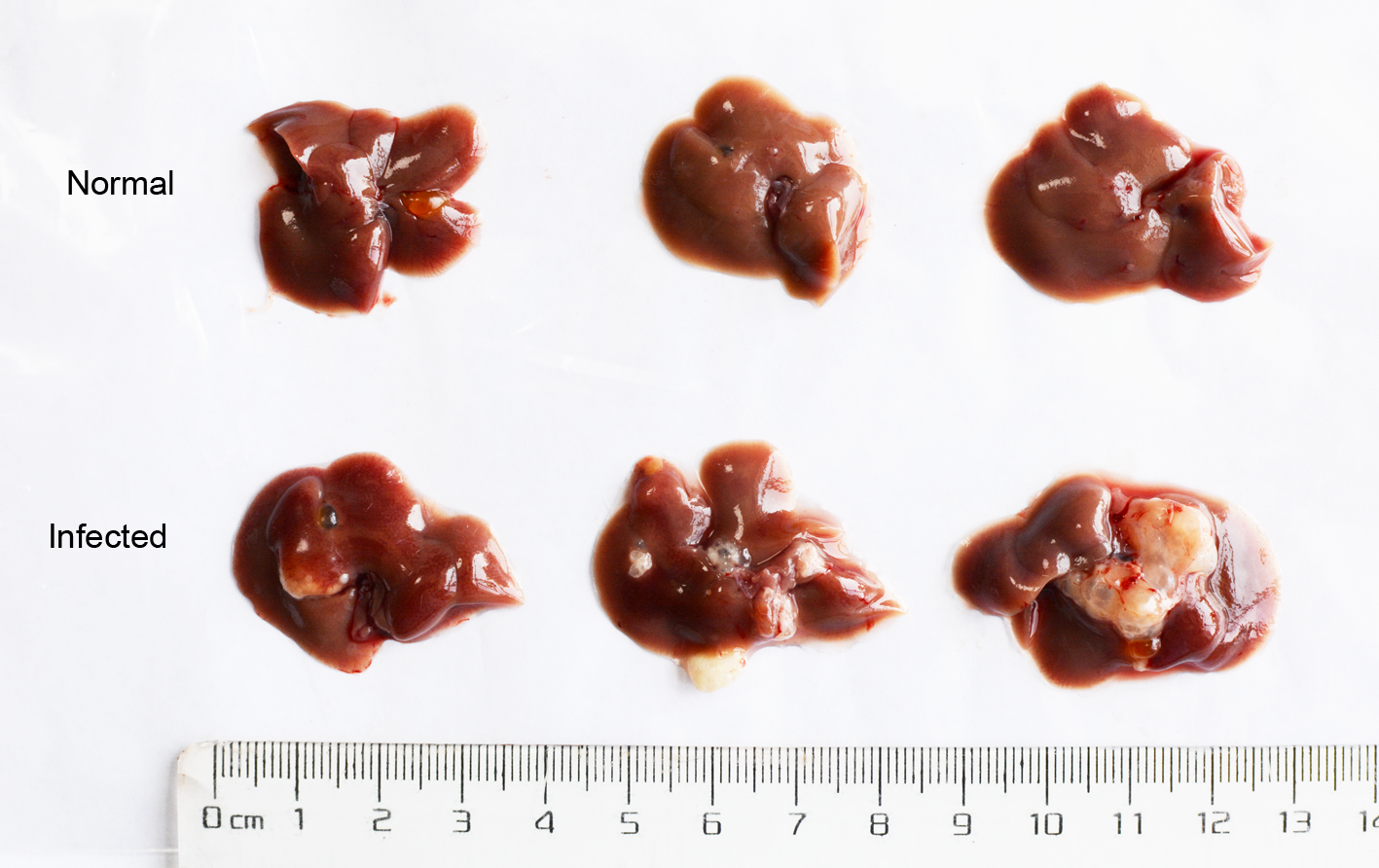

Supplement: S1 Fig — (TIF) [file pntd.0011520.s002.tif]

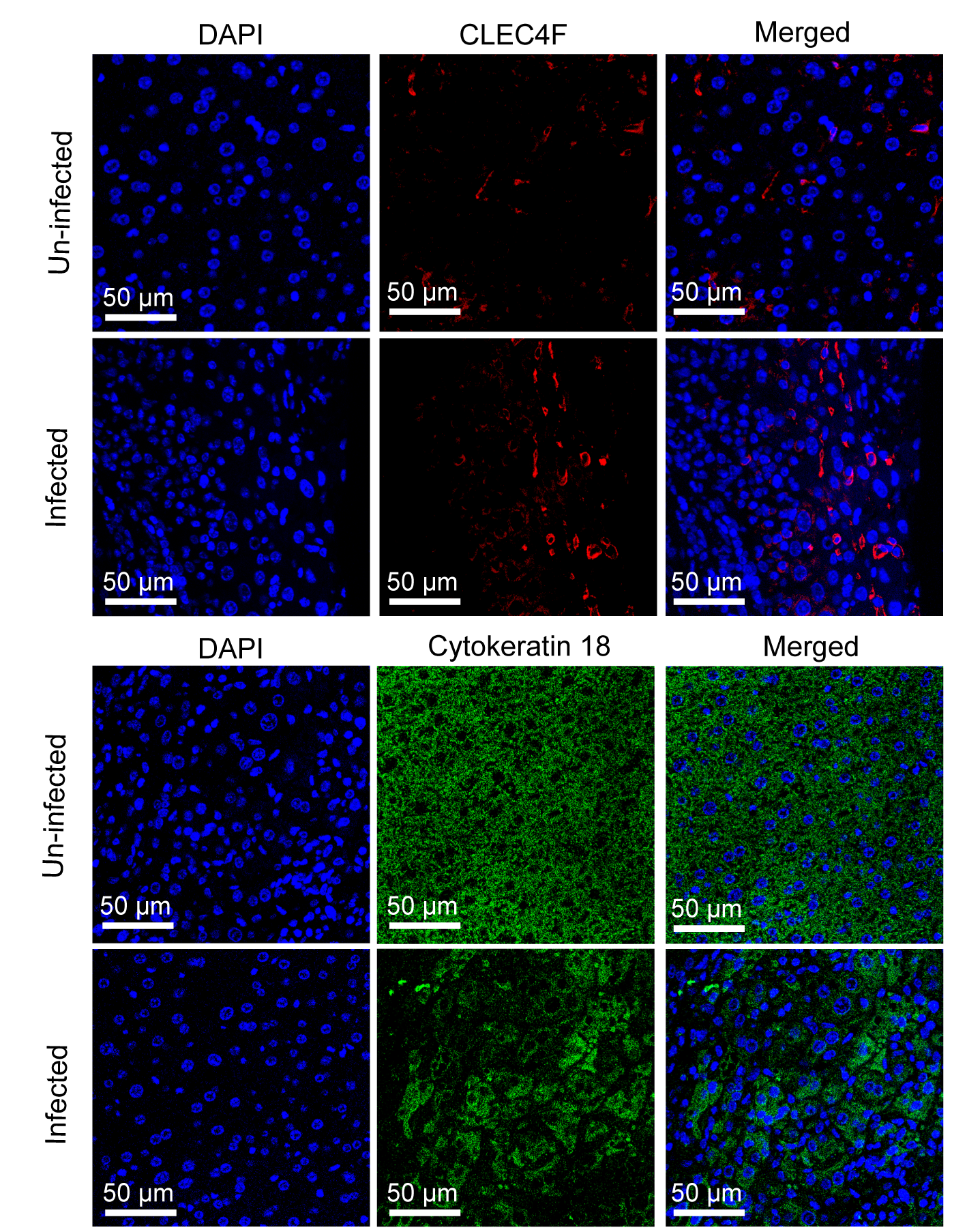

Supplement: S2 Fig — (TIF) [file pntd.0011520.s003.tif]

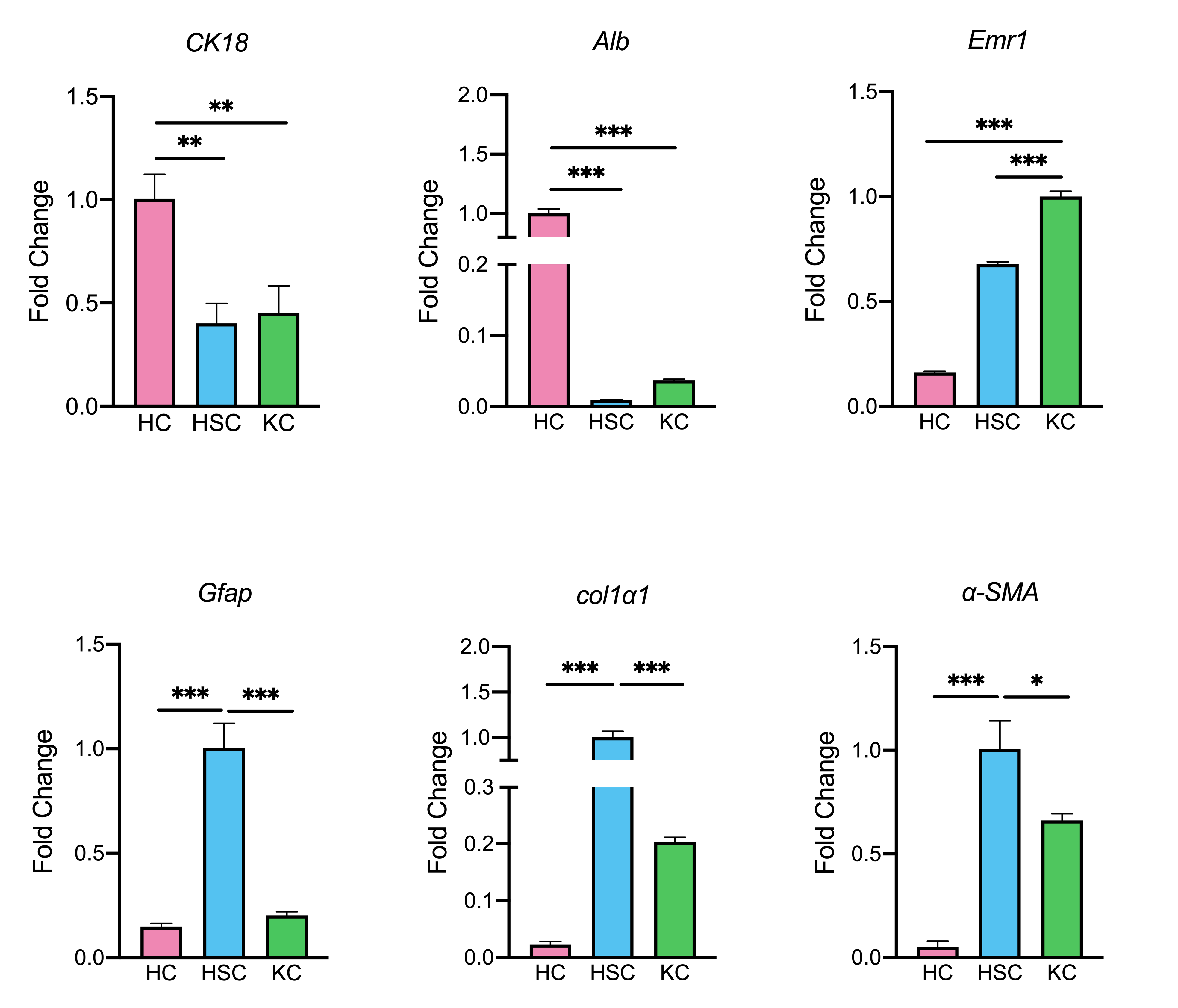

Supplement: S3 Fig — (TIFF) [file pntd.0011520.s004.tiff]
