# Supplementary material for: mmu-miRNA-342-3p promotes hepatic stellate cell activation and hepatic fibrosis induced by Echinococcus multilocularis infection via targeting Zbtb7a
Source: PLoS Negl Trop Dis. 2023 Jul 25;17(7):e0011520. doi: 10.1371/journal.pntd.0011520 (PMC10403128; doi:10.1371/journal.pntd.0011520)
Supplement: S1 Text — Table A. The qRT-PCR primers used in the study. Table B. Summary of the differentially expressed miRNAs in liver HSCs in E. multilocularis-infected mice at 60-day post infection. Table C. Summary of the differentially expressed miRNAs in liver HSCs in E. multilocularis-infected mice at 90-day post infection. Table D. Putative target genes of mmu-miR-342-3p. (DOCX) [file pntd.0011520.s005.docx]

Supplementary Material

# mmu-miRNA-342-3p promotes hepatic stellate cell activation and hepatic fibrosis induced by *Echinococcus multilocularis* infection via targeting Zbtb7a

**Shanling Cao^1,2^, Dexian Wang^1^, Yixuan Wu^1^, Junmei Zhang^1,2^,** **Lixia Pu^1^, Xuenong Luo^1^, Xueyong Zhang^1,3^, Xiaolin Sun^2^, Yadong Zheng^4,*^, Shuai Wang^1,*^, Xiaola Guo^1,*^**

*** Correspondence:**

Xiaola Guo; E-mail address: guoxiaola@caas.cn

Shuai Wang; e-mail address: wangshuai@caas.cn

Yadong Zheng; e-mail address: zhengyadong@zafu.edu.cn

**Table A. The qRT-PCR primers used in the study**

| Gene name | Catalogue |
| --- | --- |
| U6 | MmiRQP9002 |
| GAPDH | MQP027158 |
| ACTA2 | Mm20279 |
| GFAP | Mm28660 |
| Vimentin | Mm06026 |
| Col1a1 | Mm01879 |
| Mmu-miR-342-3p | MmiRQP0436 |

*These primers were purchased from GeneCopoeia

**Table B. Summary of the differentially expressed miRNAs in liver HSCs in *E. multilocularis*-infected mice at 60-day post infection.**

| **miRNA** | **Read counts** | | **log_2_(fold_change)** | ***p* value** |  |
| --- | --- | --- | --- | --- | --- |
|  | **Uninfected** | **Infected** |  |  |  |
| mmu-let-7d-3p | | 9539 | 19194 | 0.90 | 0 |
| mmu-miR-150-5p | | 35253 | 87705 | 0.90 | 0 |
| mmu-miR-92a-3p | | 13594 | 20939 | 0.54 | 0 |
| mmu-miR-497a-5p | | 22603 | 14108 | -0.58 | 0 |
| mmu-miR-27b-3p | | 26982 | 16794 | -0.59 | 0 |
| mmu-miR-27a-3p | | 103372 | 29714 | -1.22 | 0 |
| mmu-miR-33-5p | | 4106 | 1229 | -1.70 | 0 |
| mmu-miR-192-5p | | 15256 | 9039 | -0.68 | 9.29E-292 |
| mmu-miR-7a-5p | | 4891 | 8589 | 0.76 | 1.11E-261 |
| mmu-miR-744-5p | | 1099 | 76 | -3.85 | 5.90E-222 |
| mmu-miR-30c-5p | | 7939 | 4551 | -0.78 | 5.59E-171 |
| mmu-miR-322-3p | | 1708 | 3567 | 1.04 | 6.21E-165 |
| mmu-miR-199a-3p | | 13259 | 8834 | -0.54 | 1.42E-153 |
| mmu-miR-199b-3p | | 13259 | 8834 | -0.54 | 1.47E-153 |
| mmu-miR-142a-3p | | 3882 | 6054 | 0.61 | 4.23E-127 |
| mmu-miR-326-3p | | 1313 | 371 | -1.82 | 6.62E-113 |
| mmu-miR-10b-5p | | 2106 | 924 | -1.17 | 2.69E-92 |
| mmu-miR-342-3p | | 5999 | 7812 | 0.34 | 6.90E-72 |
| mmu-miR-122-3p | | 7977 | 5582 | -0.49 | 4.30E-71 |
| mmu-miR-351-5p | | 3131 | 4518 | 0.49 | 4.01E-70 |
| mmu-miR-29c-3p | | 9041 | 6646 | -0.42 | 1.05E-58 |
| mmu-miR-223-3p | | 7353 | 5249 | -0.46 | 3.79E-58 |
| mmu-miR-429-3p | | 2596 | 1530 | -0.76 | 3.25E-51 |
| mmu-miR-200a-3p | | 2226 | 1262 | -0.81 | 2.80E-50 |
| mmu-miR-142a-5p | | 6321 | 4553 | -0.46 | 2.60E-47 |
| mmu-miR-194-5p | | 5200 | 3675 | -0.48 | 5.85E-44 |
| mmu-miR-218-5p | | 763 | 304 | -1.32 | 1.25E-40 |
| mmu-miR-29b-3p | | 5574 | 4061 | -0.44 | 8.72E-39 |
| mmu-miR-872-5p | | 4746 | 3384 | -0.48 | 4.45E-38 |
| mmu-miR-139-3p | | 1982 | 2759 | 0.45 | 1.10E-36 |
| mmu-miR-31-5p | | 1020 | 501 | -1.02 | 4.79E-35 |
| mmu-miR-148a-3p | | 920 | 1424 | 0.59 | 1.14E-29 |
| mmu-miR-25-3p | | 1446 | 878 | -0.72 | 7.00E-26 |
| mmu-miR-145a-5p | | 3039 | 2152 | -0.48 | 1.06E-25 |
| mmu-miR-221-3p | | 809 | 438 | -0.87 | 1.49E-21 |
| mmu-miR-155-5p | | 1019 | 1435 | 0.48 | 1.80E-20 |
| mmu-miR-423-5p | | 1194 | 1596 | 0.41 | 1.34E-17 |
| mmu-miR-301a-3p | | 1540 | 1100 | -0.48 | 1.13E-12 |
| mmu-miR-32-5p | | 1367 | 965 | -0.50 | 3.92E-12 |
| mmu-miR-30a-3p | | 1293 | 935 | -0.46 | 5.85E-10 |
| mmu-miR-335-3p | | 396 | 566 | 0.51 | 5.16E-09 |
| mmu-miR-122b-5p | | 518 | 332 | -0.63 | 7.48E-08 |
| mmu-miR-27a-5p | | 700 | 482 | -0.52 | 1.89E-07 |
| mmu-miR-101b-3p | | 755 | 554 | -0.44 | 1.43E-05 |
| mmu-miR-31-3p | | 100 | 51 | -0.94 | 0.001 |
| mmu-miR-802-5p | | 93 | 52 | -0.83 | 0.009 |
| mmu-miR-1948-3p | | 58 | 28 | -1.03 | 0.012 |
| mmu-miR-193a-3p | | 35 | 15 | -1.22 | 0.035 |
| mmu-miR-532-5p | | 81 | 48 | -0.79 | 0.037 |

**Table C. Summary of the differentially expressed miRNAs in liver HSCs in *E. multilocularis*-infected mice at 90-day post infection.**

| **miRNA** | **Read counts** | | **log_2_(fold_change)** | ***p* value** |
| --- | --- | --- | --- | --- |
|  | **Uninfected** | **Infected** |  |  |
| mmu-miR-33-5p | 86 | 3885 | 5.445640483 | 0 |
| mmu-miR-467a-5p | 43 | 1643 | 5.230160109 | 0 |
| mmu-miR-326-3p | 46 | 1338 | 4.868993988 | 0 |
| mmu-miR-27a-3p | 6790 | 108633 | 3.204145438 | 0 |
| mmu-miR-143-3p | 6513 | 92341 | 3.087784632 | 0 |
| mmu-miR-378a-3p | 2419 | 23203 | 3.041206339 | 0 |
| mmu-miR-24-3p | 11576 | 108876 | 2.450552211 | 0 |
| mmu-miR-140-5p | 1948 | 9544 | 2.21422377 | 0 |
| mmu-miR-142a-5p | 2111 | 6788 | 1.632178022 | 0 |
| mmu-miR-151-3p | 1440 | 4120 | 1.489781746 | 0 |
| mmu-miR-223-3p | 4422 | 12142 | 1.370672885 | 0 |
| mmu-miR-652-3p | 13100 | 39752 | 1.337692719 | 0 |
| mmu-miR-30a-5p | 5082 | 10281 | 0.965920794 | 0 |
| mmu-miR-423-3p | 6052 | 11289 | 0.83908441 | 0 |
| mmu-miR-30d-5p | 14682 | 26393 | 0.71894171 | 0 |
| mmu-miR-27b-3p | 15634 | 27071 | 0.681133496 | 0 |
| mmu-miR-23a-3p | 70552 | 140442 | 0.531196193 | 0 |
| mmu-miR-22-3p | 131853 | 359685 | 0.518909334 | 0 |
| mmu-miR-29a-3p | 39445 | 25240 | -0.5249263 | 0 |
| mmu-let-7e-5p | 27070 | 15584 | -0.70382004 | 0 |
| mmu-miR-142a-3p | 12470 | 4606 | -1.34102837 | 0 |
| mmu-miR-192-5p | 9486 | 14013 | 0.514110696 | 2.77E-297 |
| mmu-miR-29b-3p | 8174 | 3864 | -1.02035959 | 4.39E-260 |
| mmu-miR-378a-5p | 581 | 2176 | 1.891534553 | 8E-249 |
| mmu-miR-194-5p | 3252 | 5880 | 0.83863022 | 1.04E-226 |
| mmu-miR-155-5p | 4286 | 1709 | -1.29958713 | 5.74E-199 |
| mmu-miR-92a-3p | 22873 | 15255 | -0.47815828 | 1.91E-194 |
| mmu-miR-328-3p | 5160 | 7880 | 0.575412147 | 4.26E-188 |
| mmu-miR-195a-5p | 5198 | 7906 | 0.580108533 | 3.48E-186 |
| mmu-miR-574-3p | 4387 | 6829 | 0.598391486 | 1.07E-173 |
| mmu-miR-322-3p | 786 | 2092 | 1.403766387 | 4.83E-163 |
| mmu-miR-125a-5p | 5462 | 2775 | -0.92210318 | 4.05E-143 |
| mmu-miR-125b-5p | 11828 | 7588 | -0.59118919 | 3.13E-125 |
| mmu-miR-3074-2-3p | 1532 | 2795 | 0.845879189 | 1.41E-110 |
| mmu-miR-10b-5p | 3511 | 1680 | -1.03704945 | 8.39E-109 |
| mmu-miR-29c-3p | 9429 | 6048 | -0.614715 | 4.22E-100 |
| mmu-let-7d-3p | 14605 | 10096 | -0.48810681 | 2.59E-99 |
| mmu-miR-484 | 2697 | 4076 | 0.577033052 | 2.25E-94 |
| mmu-miR-98-5p | 11726 | 7998 | -0.51484306 | 2.99E-87 |
| mmu-miR-350-3p | 1555 | 2598 | 0.74754188 | 3.51E-82 |
| mmu-miR-429-3p | 1046 | 1946 | 0.896227334 | 7.64E-81 |
| mmu-miR-423-5p | 2233 | 1044 | -1.08414401 | 1E-73 |
| mmu-miR-340-5p | 10309 | 7098 | -0.52120617 | 2.77E-72 |
| mmu-miR-27a-5p | 187 | 647 | 1.798366139 | 3.61E-69 |
| mmu-miR-122-3p | 7345 | 4904 | -0.55026448 | 1.08E-62 |
| mmu-miR-200a-3p | 965 | 1594 | 0.772359501 | 3.43E-49 |
| mmu-miR-450a-5p | 1768 | 897 | -0.9658764 | 4.02E-47 |
| mmu-miR-320-3p | 1295 | 606 | -1.08478636 | 5.12E-43 |
| mmu-miR-31-5p | 223 | 566 | 1.368806787 | 6.76E-42 |
| mmu-miR-335-5p | 2346 | 1361 | -0.76760619 | 7.94E-40 |
| mmu-miR-511-3p | 2481 | 1520 | -0.69850191 | 1.47E-33 |
| mmu-miR-872-3p | 906 | 1279 | 0.484966711 | 1.2E-23 |
| mmu-miR-181b-5p | 3326 | 2322 | -0.48109786 | 8.13E-22 |
| mmu-miR-101b-3p | 389 | 643 | 0.711323711 | 2.12E-20 |
| mmu-miR-338-3p | 1081 | 1368 | 0.341608254 | 2.24E-15 |
| mmu-miR-30a-3p | 1071 | 1355 | 0.343912962 | 3.22E-15 |
| mmu-miR-145a-3p | 852 | 1090 | 0.356251278 | 4.01E-13 |
| mmu-miR-30e-5p | 535 | 740 | 0.470093502 | 6.08E-13 |
| mmu-miR-214-5p | 805 | 1008 | 0.335810808 | 6.42E-11 |
| mmu-miR-450b-3p | 1546 | 1099 | -0.48581057 | 2.43E-09 |
| mmu-miR-203b-5p | 50 | 122 | 1.316027493 | 4.03E-09 |
| mmu-miR-301a-3p | 1733 | 1266 | -0.43477689 | 1.84E-08 |
| mmu-miR-744-5p | 68 | 16 | -2.04439412 | 0.000000355 |
| mmu-miR-203-3p | 643 | 440 | -0.55043863 | 0.0000201 |
| mmu-miR-130a-3p | 736 | 515 | -0.50497963 | 0.0000242 |
| mmu-miR-122b-3p | 43 | 12 | -1.84130225 | 0.000437067 |
| mmu-miR-193a-3p | 3 | 20 | 2.736965594 | 0.000520325 |

**Table D. Putative target genes of mmu-miR-342-3p**

| **Gene name** | **Gene ID** | **Gene function** | **Reference** |
| --- | --- | --- | --- |
| Agpat4  Sfn  Mmab  Lrp8  Ino80d  Fam53c  Cisd2  Zbtb7a  Plcl1  Bsn  Tcf12  Akirin1  Kcna4  Spock2 | 68262  55948  77697  16975  227195  66306  67006  16969  227120  12217  21406  68050  16492  94214 | Involved in fat metabolism  Regulate cell cycle and epidermal cell division  Regulate cobalamin metabolism  Modulates the natural immune response  Regulates cell proliferation  Transporter into the nucleus  Mitochondrial autophagy  Negative regulation TGF-β signaling pathway  Regulate lipid metabolism and signal transduction  Regulation of ubiquitinated protein ligase activity  Regulates cell differentiation  Act in Caenorhabditis elegans Development  Regulates ion transport across membranes  Regulates cell proliferation, adhesion, and invasion | [[1](#_ENREF_1)]  [[2](#_ENREF_2)]  [[3](#_ENREF_3)]  [[4](#_ENREF_4)]  [[5](#_ENREF_5)]  [[6](#_ENREF_6)]  [[7](#_ENREF_7)]  [[8](#_ENREF_8)]  [[9](#_ENREF_9)]  [[10](#_ENREF_10)]  [[11](#_ENREF_11)]  [[12](#_ENREF_12)]  [[13](#_ENREF_13)]  [[14](#_ENREF_14)] |

**References**

1. Mardian EB, Bradley RM, Aristizabal Henao JJ, Marvyn PM, Moes KA, Bombardier E, et al. Agpat4/Lpaatδ deficiency highlights the molecular heterogeneity of epididymal and perirenal white adipose depots. J Lipid Res. 2017;58(10):2037-50. Epub 2017/08/18. doi: 10.1194/jlr.M079152. PubMed PMID: 28814640; PubMed Central PMCID: PMCPMC5625126.

2. Sita G, Graziosi A, Hrelia P, Morroni F. Sulforaphane Causes Cell Cycle Arrest and Apoptosis in Human Glioblastoma U87MG and U373MG Cell Lines under Hypoxic Conditions. Int J Mol Sci. 2021;22(20). Epub 2021/10/24. doi: 10.3390/ijms222011201. PubMed PMID: 34681862; PubMed Central PMCID: PMCPMC8541491.

3. Song K, Lee HS, Jia L, Chelakkot C, Rajasekaran N, Shin YK. SMAD4 Controls Cancer Cell Metabolism by Regulating Methylmalonic Aciduria Cobalamin Deficiency (cbl) B Type. Mol Cells. 2022;45(6):413-24. Epub 2022/06/10. doi: 10.14348/molcells.2022.0067. PubMed PMID: 35680374; PubMed Central PMCID: PMCPMC9200659.

4. Omar AI, Alam MBB, Notter DR, Zhao S, Faruque MO, Thi TNT, et al. Association of single nucleotide polymorphism in NLRC3, NLRC5, HIP1, and LRP8 genes with fecal egg counts in goats naturally infected with Haemonchus contortus. Trop Anim Health Prod. 2020;52(4):1583-98. Epub 2019/12/13. doi: 10.1007/s11250-019-02154-z. PubMed PMID: 31828571.

5. Malouf GG, Su X, Yao H, Gao J, Xiong L, He Q, et al. Next-generation sequencing of translocation renal cell carcinoma reveals novel RNA splicing partners and frequent mutations of chromatin-remodeling genes. Clin Cancer Res. 2014;20(15):4129-40. Epub 2014/06/06. doi: 10.1158/1078-0432.Ccr-13-3036. PubMed PMID: 24899691; PubMed Central PMCID: PMCPMC4167829.

6. Bima AI, Elsamanoudy AZ, Alamri AS, Felimban R, Felemban M, Alghamdi KS, et al. Integrative global co-expression analysis identifies key microRNA-target gene networks as key blood biomarkers for obesity. Minerva Med. 2022;113(3):532-41. Epub 2022/03/11. doi: 10.23736/s0026-4806.21.07478-4. PubMed PMID: 35266657.

7. Huang YL, Shen ZQ, Huang CH, Lin CH, Tsai TF. Cisd2 slows down liver aging and attenuates age-related metabolic dysfunction in male mice. Aging Cell. 2021;20(12):e13523. Epub 2021/11/24. doi: 10.1111/acel.13523. PubMed PMID: 34811857; PubMed Central PMCID: PMCPMC8672792.

8. Chen L, Zhong J, Liu JH, Liao DF, Shen YY, Zhong XL, et al. Pokemon Inhibits Transforming Growth Factor β-Smad4-Related Cell Proliferation Arrest in Breast Cancer through Specificity Protein 1. J Breast Cancer. 2019;22(1):15-28. Epub 2019/04/04. doi: 10.4048/jbc.2019.22.e11. PubMed PMID: 30941230; PubMed Central PMCID: PMCPMC6438826.

9. Xiong Z, Xiao W, Bao L, Xiong W, Xiao H, Qu Y, et al. Tumor Cell "Slimming" Regulates Tumor Progression through PLCL1/UCP1-Mediated Lipid Browning. Adv Sci (Weinh). 2019;6(10):1801862. Epub 2019/05/28. doi: 10.1002/advs.201801862. PubMed PMID: 31131187; PubMed Central PMCID: PMCPMC6523368.

10. Waites CL, Leal-Ortiz SA, Okerlund N, Dalke H, Fejtova A, Altrock WD, et al. Bassoon and Piccolo maintain synapse integrity by regulating protein ubiquitination and degradation. Embo j. 2013;32(7):954-69. Epub 2013/02/14. doi: 10.1038/emboj.2013.27. PubMed PMID: 23403927; PubMed Central PMCID: PMCPMC3616282.

11. Yi S, Yu M, Yang S, Miron RJ, Zhang Y. Tcf12, A Member of Basic Helix-Loop-Helix Transcription Factors, Mediates Bone Marrow Mesenchymal Stem Cell Osteogenic Differentiation In Vitro and In Vivo. Stem Cells. 2017;35(2):386-97. Epub 2016/08/31. doi: 10.1002/stem.2491. PubMed PMID: 27574032.

12. Bowman R, Balukoff N, Clemons A, Koury E, Ford T, Baxi K, et al. Akirin Is Required for Muscle Function and Acts Through the TGF-β Sma/Mab Signaling Pathway in Caenorhabditis elegans Development. G3 (Bethesda). 2020;10(1):387-400. Epub 2019/11/27. doi: 10.1534/g3.119.400377. PubMed PMID: 31767636; PubMed Central PMCID: PMCPMC6945016.

13. Gao X, Yu S, Guan Y, Shen Y, Xu L. Nucleoporin 50 mediates Kcna4 transcription to regulate cardiac electrical activity. J Cell Sci. 2021;134(18). Epub 2021/08/20. doi: 10.1242/jcs.256818. PubMed PMID: 34409458.

14. Ren F, Wang Y, Chen P, Guo C. Downregulation of SPOCK2 promotes the proliferation, adhesion, and invasion of endometrial epithelial cells. Gynecol Endocrinol. 2021;37(3):273-7. Epub 2020/08/28. doi: 10.1080/09513590.2020.1812567. PubMed PMID: 32851893.
