# Supplementary material for: mmu-miRNA-342-3p promotes hepatic stellate cell activation and hepatic fibrosis induced by Echinococcus multilocularis infection via targeting Zbtb7a
Source: PLoS Negl Trop Dis. 2023 Jul 25;17(7):e0011520. doi: 10.1371/journal.pntd.0011520 (PMC10403128; doi:10.1371/journal.pntd.0011520)
Supplement: Attachment — Submitted filename: response to reviewers final.docx [file pntd.0011520.s006.docx]

Responses to Reviewers' Questions

**Reviewer #1:**

*The manuscript entitled "mmu-miRNA-342-3p promotes hepatic stellate cell activation and hepatic fibrosis induced by Echinococcus multilocularis infection via targeting Zbtb7a" has been reviewed. Generally, the manuscript clearly indicated the role of miRNA-342-3p in liver fibrosis associated with Echinococcus multilocularis infection.*

*The reviewer has several suggestions and comments to improve the quality of current manuscript.*

*Liver fibrosis could be caused by other* *flatworm infection such as* *Schistosoma. The authors should include related studies in section of introduction and discussion to enrich the informative manuscript for understanding the mechanism of liver fibrosis.*

Response: Thanks for your constructive comments. The following contents have been added into the introduction: “Hepatic fibrosis is a dynamic pathological process characterized by excessive deposition of extracellular matrix (ECM) during the progression of parasitic liver disease [5]. The eggs released from *Schistosoma mansoni* and *Schistosoma japonicum* primarily deposits in the periportal zones, which generate a granulomatous reaction and causing substantial pathologic liver fibrosis [7]. *Echinococcus* parasites induce an imbalance of the immune responses within the hepatic tissue, leading to the hepatic architectural distortion and the development of fibrosis [8]. The resultant liver fibrosis may be associated with severe pathology in echinococcosis with granuloma formation, collagen accumulation and inflammation [9, 10].”

*The authors globally characterized the profiles of lncRNAs-miRNAs-mRNAs in hepatocytes (HCs), Kupffer cells (KCs) and HSCs of mouse liver during E. multilocularis infection. Did they note that the Zbtb7a has different expression in different time of different type cells? The author could include the related results in the discussion.*

Response: Thank for your valuable suggestion. The expression of *Zbtb7a* was negatively correlated with the mmu-miR-342-3p expression in the HSCs from *E. multilocularis*-infected mice. There is no correlation between *Zbtb7a* and mmu-miR-342-3p in hepatocytes (HCs) and Kupffer cells (KCs). We added the related results in the discussion.

*In Fig 1A, the data indicated that the expression of Alpha-Sma has not different at 60dpi between infection and uninfection. How to explain the results?*

Response: Thanks for your comment. To further confirm this result, the expression of α-Sma at protein level was validated by Western blotting. We found that the increased expression of

α-Sma at protein level in HSCs from *E. Multilocularis-*infected mice 30-, 60-, and 90-day post infection.

*4. In Fig 5E, the manuscript is lack of the legend of fig 5E. What mean 1,2,3 in fig 5E?*

Response: Thanks for your comment, the 1, 2, and 3 in the original Fig. 5E (replaced with Fig. 6E) represent three replications in each group.

**Reviewer #2:**

*The MS by Cao et al. entitled: “mmu-miRNA-342-3p promotes hepatic stellate cell activation and hepatic fibrosis induced by* *Echinococcus multilocularis infection via targeting Zbtb7a”* *is interesting in basics but suffers from major flaws.*

*The authors state that infection induces fibrosis in liver of mice infected by EM.* *All of the message of the MS is based on this histological finding. However, this fundamental part of the analysis is badly documented:*

*In the materials and methods section there is no description how the fibrosis was* *evaluated nor by whom neither by which method.*

Response: Thanks for your constructive comments. Histologically, hepatic fibrosis in *E. Multilocularis*-infected mice was re-evaluated using hematoxylin and eosin, Masson’s trichrome, and Sirius Red staining. The collagen content in liver fibrosis was measured by the computed color image analyzer using Masson’s trichrome-stained sections, or Sirius Red-stained sections.

*Infection mice with EM is* *deleterious. In the few pics shown the authors do not show any* *metacestodes but just some portal field with fibrosis. Fibrosis develops around the metacestode and not in the portal fields. Authors have to show full blown liver lesions, measure the differences of inflammatory infiltrates, and then measure the fibrotic rim (or computer-based and with values that have been used to generate the box-plots:* *Combining Computed Tomography and Histology Leads to an Evolutionary Concept of Hepatic Alveolar Echinococcosis - PubMed (nih.gov).*

Response: Thanks for your constructive comments. As suggested, histological assessment for hepatic fibrosis was re-performed using hematoxylin and eosin, Masson’s trichrome, and Sirius Red staining. The images were taken using a light microscope (Zeiss, Germany). The full-blown liver lesions around the metacestode vesicles were shown. Images taken from 15 random 20× fields from three different liver lobes in each mouse were measured. The amount of collagen deposition was measured by the method of color thresholding segmentation using ImageJ.

Histopathological analysis revealed the excessive deposition of collagen surrounding the parasites in the liver of *E. multilocularis*-infected mice, being higher than that of uninfected mice and high-fat diet induced mice.

*Definition of stellate cells is poor. Are HSC increased during infection in situ? What is about the KCs and/or hepatocytes?*

Response: Thanks for your constructive comments. The definition of isolating HSCs from mice liver has been described in detail as follows: “To further confirm that these cells were actually HSCs, we first performed flow cytometric analysis using a HSC marker (*Glail fibrillary acidic protein*, *GFAP*). Next, Vitamin A lipid droplet autofluorescence in primary HSCs was detected at a wavelength of 328 nm using a fluorescence microscope (Zeiss, Germany). Thirdly, the expression of the biomarkers for HSCs (*α-SMA*, *GFAP* and *Col1α1*), hepatocytes (*albumin*, *Alb*), and macrophages (*EGF-like module-containing mucin-like hormone receptor-like 1*, *Emr1*) were determined by qRT-PCR.”

The results of α-SMA immunostaining showed that myofibroblastic HSCs (MF-HSCs) mainly distributed in the livers of *E. multilocularis*-infected mice around the metacestodes (Fig 2A). The proportion of MF-HSCs (α-SMA-positive cells) and macrophages (Emr1-positive cells) in the livers of *E. multilocularis*-infected mice were higher than those in un-infected group, whereas hepatocytes (Alb-positive cells) were significantly decreased (Fig 2B and S1 Fig).

*The authors must demonstrate that the effect is really based on this specific kind of infection (e.g. control groups with fat induced liver disease, or hepatitis are missing).*

Response: As suggested, the high-fat diet induced mice as control group has been added. Histopathological analysis using hematoxylin and eosin, Masson’s trichrome, and Sirius Red staining revealed the excessive deposition of collagen surrounding the metacestodes in the liver of *E. multilocularis*-infected mice, being higher than that of control mice and high-fat diet induced mice (Figs 1A and B).

*In the paper the number of experiments und number of cell cultures and repetition of experiments are not clearly given.* *This not acceptable.*

Response: Thanks for your suggestion. The number of experiments, cell cultures, and replication experiments are clearly marked in the revised manuscript.

*Minor:* *It remains a long time unclear whether these experiments have been performed in mice or humans. Authors should consider to shift the experiments in the human system. This is the major health problem, not mice. Cultures of Human stellate cells are available.*

Response: Thanks for your constructive comment. We will perform those experiments on human stellate cells or liver tissues of AE.

If all this major issue are taken in account I am willing to re-review the MS.

Response: Thank you for considering the revised version of our manuscript. We have thoughtfully taken into account these comments. Point-by-point responses to all the comments are provided above.

**Reviewer #3:**

*I think this paper miRNA-342-3p promotes HSC activation induced by E. multilocularis infection is very important and* *novel.*

*Major issues:*

*A copy of ethical approval is needed*.

Response: Thanks for your comment. The copy of ethical approval has been provided as attachment when we submit the revised manuscript.

*In line 78, it’s better to mention the total number of mice used. And how it’s grouped because here you mentioned you divided them into 2 group, then in line 98 you said that “Relative expression levels of miRNAs in three groups” ???!*

Response: Thanks for your constructive comments. The subsection has been re-written: “Four-week-old BALB/c mice (n= 160) were randomly divided into two groups. One group (n= 80) was intraperitoneally injected with 600 *E. multilocularis* protoscoleces, which were obtained from the hydatid cysts in infected mouse in our lab as previously described [23]. The other (n= 80) was inoculated with 0.9% saline solution as an uninfected group.

We revised as follows: “Relative expression levels of miRNAs in the uninfected and *E. multilocularis*-infected groups at different time points were analyzed using DESeq R package (1.8.3) and differentially expressed miRNAs were identified.”

I*n line 83, more details about the method of isolating HSCs from mice liver is needed.*

Response: Thanks for your constructive comments. The method of isolating HSCs from mice liver has been described in detail as follows: “Primary HSCs were isolated from uninfected (n=6) and infected (n=6) mouse liver by collagenase Ⅳ perfusion, followed by density gradient centrifugation as previously described [24]. Briefly, the livers were perfused with 0.09% EGTA buffer at a flow rate of 5 mL/min at 42°C for 2 min and then with Enzyme buffer containing 0.04% collagenase at a flow rate of 5 mL/min at 42°C for 6 min Ⅳ. The resultant digested livers were excised, and *in vitro* digestion was performed in 80 mL Enzyme buffer containing 0.08% collagenase Ⅳ and 1% DNase at 37°C for 30 min. The cells were passed through nylon filters (70 μm) and centrifuged at 50 g at 4°C for 4 min. The supernatant was centrifuged at 600 g for 10 min, and the pellet was washed by Gey’s balanced salt solution (GBSS) at 500 g at 4°C for 5min to obtain nonparenchymal cells (NPCs). The NPCs were resuspended in 5 mL GBSS and gently coated with Optiprep solutions of different concentrations, with 8 mL 11.5% Optiprep in the upper layer and 4 mL 20% Optiprep in the lower layer, and centrifuged uninterrupted at 1,400 ×g at 4°C for 17 min. The upper cells (HSCs) were transferred to tubes and washed three times with GBSS. Cells were cultured in DMEM medium containing 10% FBS for subsequent experiments.

For further validation, we first performed flow cytometry analysis using a HSC marker (*Glail fibrillary acidic protein*, *GFAP*). Next, Vitamin A lipid droplet autofluorescence in isolated primary HSCs was detected at a wavelength of 328 nm using a fluorescence microscope (Zeiss, Germany). Thirdly, the expression of the biomarkers for HSCs (*α-SMA*, *GFAP* and *Col1α1*), hepatocytes (*albumin*, *Alb*), and macrophages (*EGF-like module-containing mucin-like hormone receptor-like 1*, *Emr1*) were determined by qRT-PCR.

Primary HSCs were cultured in DMEM medium containing 10% FBS, and transfection experiments were performed (at 5 days after isolation). HSCs (5 × 10^5^ cells per well) were plated into 12-well plates and transfected with 50 nM mmu-miR-342-3p inhibitor or 50 nM negative control (NC) (Ambion/Invitrogen) using Lipofectamine™ RNAiMAX Transfection Reagent (Invitrogen). Following transfection for 10 h, the medium was replaced with fresh DMEM medium supplemented with 10% FBS. Each transfection was independently repeated three times.

*In line 133, the authors had mentioned that the liver was fixed in* *4% paraformaldehyde, is the paraformaldehyde is prepared in Phosphate buffer saline or what, and is this protocol for immunochemistry does not require antigen retrieval?*

Response: Yes, the 4% paraformaldehyde is prepared in Phosphate buffer saline. The antigen retrieval was performed in this protocol for immunochemistry. We added in the revised manuscript.

Minor Issues:

*Clarification of abbreviations is needed to provide better understanding especially for those who are not very familiar with the field of the study.*

Response: Thanks for your suggestion. The abbreviation has added in the revised manuscript.
