# Supplementary material for: mmu-miRNA-342-3p promotes hepatic stellate cell activation and hepatic fibrosis induced by Echinococcus multilocularis infection via targeting Zbtb7a
Source: PLoS Negl Trop Dis. 2023 Jul 25;17(7):e0011520. doi: 10.1371/journal.pntd.0011520 (PMC10403128; doi:10.1371/journal.pntd.0011520)
Supplement: Attachment — Submitted filename: Responses to Reviewer.docx [file pntd.0011520.s008.docx]

Reviewer's Responses to Questions
*Reviewer #2: Improved. However, as I recommeded in my first review I ask the authors again to insert a histological figure of a full blown liver lesion of a mouse after infection as a proof of principle. Up to now, the authors show histology of mice with non characteristic periportal fibrosis; to insert a histological figure of the charcteristic liver lesion after infection is an item that should be easily done since infection rates are high (as have stated the authors); to solve this point an additional supplemental figure is enough. The authors have not answered to this point raised in my previous review.*
Response: Thanks for your constructive comments. As suggested, figures of the full-blown liver lesions after infection were added in S1 Fig.
*Figure Files:*

*While revising your submission, please upload your figure files to the Preflight Analysis and Conversion Engine (PACE) digital diagnostic tool, https://pacev2.apexcovantage.com. PACE helps ensure that figures meet PLOS requirements. To use PACE, you must first register as a user. Then, login and navigate to the UPLOAD tab, where you will find detailed instructions on how to use the tool. If you encounter any issues or have any questions when using PACE, please email us at figures@plos.org.*

Response: Confirmed.

*Data Requirements:*

*Please note that, as a condition of publication, PLOS' data policy requires that you make available all data used to draw the conclusions outlined in your manuscript. Data must be deposited in an appropriate repository, included within the body of the manuscript, or uploaded as supporting information. This includes all numerical values that were used to generate graphs, histograms etc.. For an example see here: http://www.plosbiology.org/article/info%3Adoi%2F10.1371%2Fjournal.pbio.1001908#s5.*

Response: Confirmed.

*References:*

*Please review your reference list to ensure that it is complete and correct. If you have cited papers that have been retracted, please include the rationale for doing so in the manuscript text, or remove these references and replace them with relevant current references. Any changes to the reference list should be mentioned in the rebuttal letter that accompanies your revised manuscript. If you need to cite a retracted article, indicate the article's retracted status in the References list and also include a citation and full reference for the retraction notice.*

Response: Confirmed.
