# Supplementary material for: mmu-miRNA-342-3p promotes hepatic stellate cell activation and hepatic fibrosis induced by Echinococcus multilocularis infection via targeting Zbtb7a
Source: PLoS Negl Trop Dis. 2023 Jul 25;17(7):e0011520. doi: 10.1371/journal.pntd.0011520 (PMC10403128; doi:10.1371/journal.pntd.0011520)
Supplement: Attachment — Submitted filename: Responses to Reviewer.docx [file pntd.0011520.s009.docx]

Responses to Reviewer

*While reviewing the revised manuscript, I have concluded that all comments and criticisms presented by the reviewers have been correctly addressed. However, I have also noticed that in Fig. 5A, the graph shows that the miRNA mimic increased luciferase activity, while the text indicates that luciferase activity was decreased by the RNA mimic (as would be expected, given the rest of the results shown in Figs. 5 and 6). Please check if there was an error during the preparation of Fig. 5A, or revise the text and conclusions if necessary.*

Response: Thanks for your comment. We have checked and confirmed the raw data. It is our mistake to make the graph. We have revised it in Fig 5A.

*Additionally, in Fig. 5A the inset reads "Mimcs" instead of "mimic", and the NC mimic is not described in the text or figure legend.*

Response: Thanks for your comment. We have replaced Mimcs to mimic in Fig 5A. The definition of NC or mimic has added in the figure legend.

*Finally, please check l. 258, "hepatocytes (Alb-positive cells) were significantly decreased (Fig 2B and S2 Fig).", as the marker for hepatocytes used in Fig. S2 was cytokeratin 18, and not albumin.*

Response: Thanks for your comment. The albumin and cytokeratin 18 are both markers of hepatocytes. Albumin is usually used for mRNA expression level detection and cytokeratin 18 is usually used for protein level detection. As suggested, we have added the mRNA expression level of cytokeratin 18 in S3 Fig.

*Figure Files: While revising your submission, please upload your figure files to the Preflight Analysis and Conversion Engine (PACE) digital diagnostic tool, https://pacev2.apexcovantage.com. PACE helps ensure that figures meet PLOS requirements. To use PACE, you must first register as a user. Then, login and navigate to the UPLOAD tab, where you will find detailed instructions on how to use the tool. If you encounter any issues or have any questions when using PACE, please email us at figures@plos.org.*

Response: Confirmed.

*Data Requirements:*

*Please note that, as a condition of publication, PLOS' data policy requires that you make available all data used to draw the conclusions outlined in your manuscript. Data must be deposited in an appropriate repository, included within the body of the manuscript, or uploaded as supporting information. This includes all numerical values that were used to generate graphs, histograms etc.. For an example see here: http://www.plosbiology.org/article/info%3Adoi%2F10.1371%2Fjournal.pbio.1001908#s5.*

Response: Confirmed.

*Reproducibility:*

*To enhance the reproducibility of your results, we recommend that you deposit your laboratory protocols in protocols.io, where a protocol can be assigned its own identifier (DOI) such that it can be cited independently in the future. Additionally, PLOS ONE offers an option to publish peer-reviewed clinical study protocols. Read more information on sharing protocols at* [*https://plos.org/protocols?utm_medium=editorial-email&utm_source=authorletters&utm_campaign=protocols*](https://plos.org/protocols?utm_medium=editorial-email&utm_source=authorletters&utm_campaign=protocols)

Response: Confirmed.

*References:*

*Please review your reference list to ensure that it is complete and correct. If you have cited papers that have been retracted, please include the rationale for doing so in the manuscript text, or remove these references and replace them with relevant current references. Any changes to the reference list should be mentioned in the rebuttal letter that accompanies your revised manuscript. If you need to cite a retracted article, indicate the article's retracted status in the References list and also include a citation and full reference for the retraction notice.*

Response: Confirmed.
